# Urinary Bladder Cancer in Egypt: Are There Gender Differences in Its Histopathological Presentation?

**DOI:** 10.1155/2018/3453808

**Published:** 2018-03-13

**Authors:** Fiorina Kyritsi, Christopher A. Loffredo, Yun-Ling Zheng, George Philips, Sania Amr

**Affiliations:** ^1^Lombardi Comprehensive Cancer Center, Georgetown University, Washington, DC, USA; ^2^University of Maryland School of Medicine, Baltimore, MD, USA; ^3^Marlene and Stewart Greenebaum Comprehensive Cancer Center, University of Maryland, Baltimore, MD, USA

## Abstract

We investigated gender differences in the histopathologic presentation of bladder cancer cases in Egypt, where both urothelial cell carcinoma (UC) and squamous cell carcinoma (SCC) types are highly prevalent. We used logistic regression to estimate the unadjusted (OR) and adjusted odds ratio (AOR) and 95% confidence interval (CI) of the associations between gender and different histopathologic and sociodemographic parameters of 2,186 confirmed cases of primary bladder cancer (1,775 males and 411 females; 784 SCC and 1,402 UC). There were no statistically significant gender differences in tumor grade, stage, mucosal ulcer, or inflammatory cystitis, regardless of the cancer type, but men were less likely than women to have undergone cystectomy with pelvic lymphadenectomy. Having *Schistosoma haematobium* (*SH*) ova in the bladder tissue was significantly associated with male gender in the fully adjusted model of either SCC (AOR (95% CI) = 2.12 (1.15–3.89)) or UC cases (3.78 (1.89–7.55)). Compared to females, male cases were significantly older at time of diagnosis and smokers. In Egypt, regardless of the type of bladder cancer (SCC or UC), male more than female cases had evidence of *SH* infection, but not other histopathologic differences, in bladder tissue specimens.

## 1. Introduction

Worldwide, the incidence and prevalence of urinary bladder cancer are higher in men than in women [1], whereas several studies, reviewed by Fajkovic et al., have shown worse outcomes after radical cystectomy in the latter than in the former [2]. Indeed, to predict recurrence of bladder cancer after surgery, the International Bladder Cancer Nomogram Consortium (IBCNC) developed a tool that includes, among other variables, sex, cancer histological type, and characteristics, as prognostic factors [3]. However, Welty et al. developed a nomogram to predict survival after cystectomy for urothelial cell carcinoma, but did not include sex, because they found age and tumor stage to be considerably stronger risk factors than sex in predicting patient survival [4].

Previous studies of the mechanisms underlying the variance in prognosis between men and women following bladder cancer diagnosis led researchers to suggest differences in exposures to carcinogens, hormones, and/or cellular and physiologic differences [5]. Known risk factors for bladder cancer include tobacco smoke, occupational exposure to aromatic amines and polycyclic aromatic hydrocarbons, and chronic urinary tract infections with *Schistosoma haematobium* (*SH*) [6–9].

The gender difference in this malignancy's incidence was at one time attributed to higher exposure to tobacco and occupational carcinogens in men than in women [10]. However, these risk factors failed to account fully for the gender disparity [11]. Other investigators focused on sex hormones and reproductive factors in searching for an explanation for the elevated bladder cancer risk among men. Both animal models [12, 13] and epidemiological studies showed evidence supporting the postulate that estrogens [14–18] and antiandrogens [19] are protective against this malignancy, particularly the urothelial type. Indeed, there are two main histological types of urinary bladder cancer, urothelial cell (UC) and squamous cell carcinoma (SCC). The latter, prevalent in areas endemic for *SH* infection, is rare (5%) in Europe and the United States, where the former is the predominant type (90%) on which most of the studies were conducted.

In their quest for understanding the gender disparity in bladder cancer incidence and outcomes after treatment, investigators focused on the clinical presentation, diagnosis, and histopathological characteristics of the bladder tumors [20, 21]. Delayed referral and diagnosis in women resulted in more advanced stage of the tumor and hence the negative outcomes following cystectomy [22, 23]. Some investigators reported high-grade and aggressive type of tumor as the reason for worse outcomes in women [24], but not others [25]. Not only were the results of these studies conflicting, and thus not conclusive, but also they involved predominantly the UC type of bladder cancer.

In Egypt, where bladder cancer is the second most common malignancy among men and 30% of the cases are SCC type, we investigated the gender differences for environmental bladder cancer risk factors separately for each of the histological types [26, 27]. In the present study, we describe the histopathological presentation of the two main types (UC and SCC) and their gender differences.

## 2. Materials and Methods

The present investigation used data collected on primary urinary bladder cancer in Egypt for the parent study, a multicenter case-control type that was conducted between 2006 and 2014. It was approved by the institutional review boards of the three collaborating cancer centers in Egypt: the University of Maryland in Baltimore MD, Georgetown University in Washington DC, and the National Scientific and Research Ethical Committee at the Egyptian Ministry of Health and Population.

### 2.1. Study Population

Bladder cancer cases were recruited from the National Cancer Institute in Cairo, the Minia Oncology Center in Minia, and the South Egypt Cancer Institute in Assiut. Participants had to be (i) adults between the ages of 19–80 years, (ii) self-identified as physically able to participate in an interview, and (iii) diagnosed with presumed urinary bladder cancer within 12 months. Individuals who had a prior history of other malignancies were excluded from the study [18, 26–28].

### 2.2. Questionnaire

After explaining the study and obtaining informed consent, trained interviewers administered a structured questionnaire to consented participants and collected information about sociodemographic characteristics and occupational and medical histories including the history of schistosomiasis. History of smoking and/or being exposed to secondhand smoke was documented.

### 2.3. Case Ascertainment

The study pathologists reviewed the pathology reports, the available surgical or biopsy specimen slides, and/or the referred slides for each urinary bladder cancer case. They first classified cases as (1) papillary transitional cell carcinoma (TCC), (2) invasive TCC, (3) squamous cell carcinoma (SCC), (4) adenocarcinoma, or (5) others, including undifferentiated carcinomas. Cases of carcinoma that had metastasized to the urinary bladder were excluded from the study. The cases of TCC were classified based on the malignancy presentation as papillary or flat and whether it is confined to the transitional epithelium or invading the other layers of the bladder wall. All TCC cases were grouped and labeled as urothelial cell carcinoma (UC). The numbers of adenocarcinomas and other malignancies were so small, and thus not included in the present study.

### 2.4. Histopathological Characteristic Variables

Depending on the type of specimen examined, the pathologist noted the tumor grade (from 1 to 3), the stage (from p0 to pIVb), and the presence of *SH* ova. In addition, the pathologist examined the nonneoplastic bladder tissue, whenever available, for signs of inflammatory cystitis and/or mucosal ulcer.

### 2.5. Variables of Interest

Gender (male, female) was the main variable of interest with the reference group being female for statistical comparisons. Smoking was categorized as none (if they had smoked less than 100 cigarettes in their lifetime and had never smoked a waterpipe), waterpipe only, cigarettes only, and both (waterpipe and cigarettes). Smoking was also dichotomized as ever versus never to include in some of the analyses. The number of pack-years of cigarettes smoked by smokers and former smokers was calculated and added in any analysis that included the smoking variable. Participants, who reported ever-been told of a diagnosis of schistosomiasis by their doctors, were classified as having positive history of the disease. Secondhand smoke exposure was categorized as none or exposure either at home, outside the home, or at work. Education was categorized as none versus some (as a large number of the cases were uneducated). Similarly, the majority was married, and hence, marital status was either yes or no; the latter included never married, divorced, widowed, or separated. Residence location was categorized as north (Cairo, Giza, and northward) versus south of Cairo and urban versus rural. We grouped age in categories for descriptive analysis and dichotomized it (≤55 years versus >55 years) for the multivariable analysis.

### 2.6. Statistical Analysis

We used, respectively, Student's *t*-test and *χ*^2^ tests to compare continuous and categorical variables between males and females, separately for UC and SCC. Significance level was set at *p* ≤ 0.05. We used logistic regression to estimate unadjusted (OR) and adjusted odds ratio (AOR) and 95% confidence interval (CI). We used SAS software 9.4 (SAS Institute Inc., NC) for all analyses.

## 3. Results

From July 2006 through July 2014, 4,049 patients presumed to have bladder cancer were asked to participate in the study. Of those patients, 3,427 were eligible and 2,891 (84%) agreed to participate. The pathologists completed a review of 2,523 cases and confirmed 2,325 to be primary bladder cancer. There were 1,402 UC (60.3%), 784 SCC (33.7%), 77 adenocarcinomas (3.3%), and 62 (2.7%) cases of other types of primary bladder cancer.

Table 1 shows the sociodemographic characteristics of the SCC and UC cases separately and by gender. The mean age at diagnosis of bladder cancer was higher for men than that for women (57.2 years versus 53.4 years, *p* value < 0.0001; 61.2 years versus 59.2 years, *p* value 0.01) for SCC and UC, respectively. Very few women smoked or had some education. The majority of participants were married. Whether the cases were SCC or UC, more men (55%) than women (20%) reported having a history of schistosomiasis.

Table 2 shows the histopathological characteristics of SCC and UC separately by gender.

The men-to-women ratios were 2.7 and 5.9 for SCC and UC, respectively. Among the SCC cases, we found a significant difference (*p*=0.01) between men and women in the prevalence of cystectomy; pelvic lymphadenectomy was used among women but not men for both types of malignancies. The distribution of UC types (papillary and invasive) among men and women was similar, with the majority (77.8% and 78.8%, resp.) being invasive. In both men and women, the tumor grade was predominantly 2 for SCC (62% and 70% of the cases) and 3 for UC (49%). There were no significant differences between men and women in the distribution of their tumor stages; however, more SCC (∼87% of the cases for both genders) than UC (59% and 57% of the men and women cases, resp.) were stage II and above.

The presence of *SH* ova in the tumor specimen was significantly more prevalent among men (73% and 59% for SCC and UC, resp.) than among women (53% and 42% for SCC and UC, resp.) (*p* < 0.0001). This pattern was consistent with the self-reported history of schistosomiasis, but with a greater magnitude.

Based on the pathological stage (stage II and above), muscle invasion was noted in ∼87% of SCC cases but only in 57–59% of UC cases, with no significant gender difference. There was no gender difference for either inflammatory cystitis and/or ulcer in the nonneoplastic bladder tissue specimen examined, although both signs were highly prevalent in both types of carcinoma (Table 2).

Considering the small number of subjects with grade 1, we combined it with grade 2 and compared them to grade 3 in the subsequent analysis.  Table 3 shows the results of the unadjusted associations between male gender and different relevant variables, as well as the results of the multivariable logistic regression analyses, for SCC and UC cases, separately. We included in the final model variables that were significantly associated with male gender. Among the SCC cases, having high-grade tumor (grade 3) was significantly associated with male gender (OR (95% CI) = 1.64 (1.06–2.54)), but the association became statistically nonsignificant (0.82 (0.38–1.794)) after adjustment for other significant variables. On the other hand, having *SH* ova in the bladder tissue specimen (2.12 (1.15–3.89)), older age (2.40 (1.32–4.36)), smoking (24.89 (8.58–72.21)), and residing in the south (3.31 (1.14–9.69)) remained significantly associated with male gender in the adjusted model (Table 3).

Among the UC cases, neither tumor grade nor residence in the south, but having *SH* ova in the bladder tissue (3.78 (1.89–7.55)), smoking (40.55 (9.13–180.12)), and older age (3.44 (1.73–6.83)) were associated with male gender; the associations were similar to those noted among the SCC cases (Table 3).

## 4. Discussion

We found male, more than female, cases of bladder cancer to have evidence of *SH* infection in their bladder tissue specimen, regardless of the type of cancer. These findings support our previous studies demonstrating that the self-reported history of *SH* infection is a risk factor, not only for SCC but also for the UC cancer type [26, 28]. Indeed, more men (55%) than women (20%) reported a history of schistosomiasis, and the pathologists reported more men (73% and 59% for SCC and UC, resp.) than women (53% and 42% for SCC and UC, resp.) to have *SH* ova in their bladder tissue specimens. The higher prevalence of *SH* infection in males, as compared to female cancer cases, can be explained by the fact that the majority of men worked in farming and thus were at risk for *SH* infection because of exposures to infested waters, while the majority of women did not work outside the house [28].

We found no gender differences in the grade and the stages of either SCC or UC; however, the male-to-female ratio was higher for UC (5.9) than for SCC (2.7). In addition, most men in this study were smokers, while few women reported smoking. It is probable that the addition of *SH* infection to smoking, which is a well-established risk factor for UC, contributed to the male-to-female ratio being higher for UC than for SCC, among bladder cancer cases in Egypt.

Bladder cancer is one of the few malignancies where women have high cancer-specific mortality [22]. One of the reasons suggested was gender differences in stage distribution [23]; the latter being worse in female than in male patients [24]. We found men and women to have similar distribution of their tumor stages, whether it was UC or SCC (Table 2). High-grade tumor and thus aggressive malignancy were also suggested to occur among women more than men. We found that grade 3 SCC, but not UC, was associated with male, not female, gender; however, the association became nonsignificant after adjustment for other known risk factors (Table 3). A prior study of UC reported no gender differences in the histopathological characteristics of the tumor [25]. And more recently, Welty et al. developed a new nomogram to predict UC survival following surgery, based on the tumor characteristics, and did not include sex as a prognostic factor [4]. Our study is the first to investigate gender differences in the grade or the stage of the SCC type.

Investigators found that muscle-invasive cases occurred more frequently in men than in women, and those men were younger than the women at diagnosis [21]. In our study, we found no gender differences in the prevalence of muscle invasion: although muscle invasion was more prevalent in the SCC (∼87%) than in the UC (57–59%) cases, women cases were diagnosed with bladder cancer at a significantly younger age than men, particularly with the SCC type. A Turkish study found 75% of the bladder cancer cases to be nonmuscle invasive [29].

We found that women, more commonly than men, underwent cystectomy with pelvic lymphadenectomy. This is consistent with a diagnosis made at advanced stages of the disease. However, in our study, women cases who underwent radical cystectomy were comparable to those who underwent pelvic lymphadenectomy, with respect to the grade and stage of the malignancy. Possible explanations include the existence of comorbidities leading to pelvic lymphadenectomy or differences in surgical practices between clinicians. Indeed, comparing the three referral cancer centers, Cairo NCI, Minia, and Assiut, we found that, among all women who underwent cystectomy, pelvic lymphadenectomy represented 30%, 67%, and 43% of the cases, respectively.

The search for factors associated with gender differences in bladder cancer incidence has prompted several investigators to assess the role of sex steroids. In animal studies, androgen receptor knockout (AR-KO) male mice developed fewer bladder tumors, and females without AR developed no tumors [13, 14]. Additionally, there is evidence that the presence of female hormones can inhibit bladder carcinogenesis or delay the disease progression [15], and the use of antiandrogens appears to delay the development of bladder cancer in men [19]; all of these lines of evidence support a theory that female hormones are protective against bladder cancer. Some investigators and we [16, 18] found estrogen to be protective but others did not [17]. Further studies are needed to assess the contribution of sex steroids to the gender disparity of bladder cancer in Egypt.

Our study has some weaknesses. Some of the information collected was self-reported and thus subject to bias. Despite the large sample size, very few women reported ever-smoking tobacco, and therefore, the risk estimates may not be precise. Nonetheless, our study has several strengths, such as a large sample size that included both UC and SCC cases and a large number of women with SCC, which allowed us to examine for gender-specific differences in histopathological presentation of this type of malignancy. It is a multicenter study, which recruited cases from several areas in Egypt, and it has a high participation rate (84%). Finally yet importantly, two pathologists used a standardized form to report the histopathological characteristics and ascertain the type of the tumors.

## 5. Conclusion

We found evidence for bladder tissue infection with *SH* to be significantly more prevalent in male than in female cases of bladder cancer, regardless of the type of cancer (SCC or UC); but there are no significant gender differences in other histopathological characteristics of either UC or SCC in Egypt. More studies are needed to elucidate the mechanism(s) underlying gender difference in bladder cancer.

## Figures and Tables

**Table 1:** Sociodemographic characteristics of bladder cancer cases (squamous cell carcinoma (SCC) and urothelial cell carcinoma (UC)) in Egyptian men and women.

Variable	Males		Females		^∗^ *p* value
	SCC (*N*=574)	UC (*N*=1201)	SCC (*N*=210)	UC (*N*=201)	
Age ± SD (years)	57.2 ± 10.3	61.2 ± 10.3	53.4 ± 10.8	59.2 ± 11.1	SCC < 0.0001; UC 0.01
Age group (years)					
≤45	64 (11.1)	89 (7.4)	49 (23.4)	24 (11.9)	
>45–≤55	182 (31.7)	259 (21.6)	83 (39.5)	46 (22.9)	SCC < 0.0001
>55–≤65	206 (35.9)	421 (35.0)	53 (25.2)	73 (36.3)	UC 0.06
>65	122 (21.3)	432 (36.0)	25 (11.9)	58 (28.9)	
Marital status					
Not married	11 (1.9)	11 (0.9)	7 (3.3)	1 (0.5)	SCC 0.2
Married	563 (98.1)	1190 (99.1)	203 (96.7)	200 (99.5)	UC 0.5
Education					
None	458 (79.8)	804 (67.0)	193 (91.9)	177 (88.1)	SCC < 0.0001
Some	116 (20.2)	395 (33.0)	17 (8.1)	24 (11.9)	UC < 0.0001
Residence					
North	36 (6.3)	176 (14.7)	27 (12.9)	28 (13.9)	SCC 0.002
South	538 (93.7)	1025 (85.3)	183 (87.1)	173 (86.1)	UC 0.6
Urban	52 (9.1)	217 (18.1)	21 (10.0)	35 (17.4)	SCC 0.6
Rural	522 (90.9)	984 (81.9)	189 (90)	166 (82.6)	UC 0.8
Smoking					
None	99 (17.3)	144 (12.0)	203 (96.6)	196 (97.5)	
Waterpipe	80 (13.9)	121 (10.1)	4 (2.0)	2 (1.0)	SCC < 0.0001
Cigarette	322 (56.1)	761 (63.3)	3 (1.4)	3 (1.5)	UC < 0.0001
Both	73 (12.7)	175 (14.6)	0	0	
Schistosomiasis					
No	211 (36.8)	469 (39.0)	148 (70.5)	145 (72.1)	SCC < 0.0001
Yes	320 (55.7)	670 (55.8)	44 (20.9)	41 (20.4)	UC < 0.0001
Unknown	43 (7.5)	62 (5.2)	18 (8.6)	15 (7.5)	
Secondhand smoke					
No	46 (8.0)	92 (7.7)	5 (3.5)	3 (2.5)	SCC 0.06
Yes	527 (92.0)	1106 (92.3)	139 (96.5)	119 (97.5)	UC 0.03

^∗^Comparison of *p* value between males and females was done using Student's *t*-test and *χ*^2^ for continuous and categorical variables, respectively. For some variables, the sum does not add up to the total because of missing information.

**Table 2:** Histopathological characteristics of bladder cancer cases (squamous cell carcinoma (SCC) and urothelial cell carcinoma (UC)) in Egyptian men and women.

Variable	Males		Females		*p* value^1^
	SCC (*N*=574)	UC (*N*=1201)	SCC (*N*=210)	UC (*N*=201)	
Referred slide	28 (4.9)	66 (5.5)	9 (4.3)	14 (7.0)	
Biopsy	292 (51.1)	786 (65.9)	83 (40.4)	140 (69.6)	UC 0.4
Cystectomy	251 (44.0)	340 (28.5)	114 (54.8)	47 (23.4)	SCC 0.01
Radical	223 (95.3)	268 (94.0)	49 (51.1)	21 (52.5)	
Simple	7 (3.0)	7 (2.5)	1 (1.0)	0	
Partial	4 (1.7)	9 (3.2)	7 (7.3)	0	SCC < 0.0001
Pelvic excision^2^	0	0	39 (40.6)	19 (47.5)	UC < 0.0001
Grade					
1	84 (15.0)	55 (4.8)	31 (15.2)	7 (3.7)	SCC 0.07
2	353 (62.9)	527 (46.2)	143 (70.1)	89 (47.1)	UC 0.8
3	124 (22.1)	558 (49.0)	30 (14.7)	93 (49.2)	
Stage					
0	0	38 (6.7)	1 (0.8)	6 (7.6)	
I	41 (12.8)	195 (34.3)	15 (11.6)	28 (35.4)	
IIa	22 (6.9)	19 (3.3)	8 (6.2)	4 (5.0)	SCC 0.6
b	82 (25.7)	72 (12.6)	29 (22.5)	10 (12.7)	UC 0.6
IIIa	33 (10.3)	54 (9.5)	12 (9.3)	4 (5.0)	
b	115 (36.0)	144 (25.3)	54 (41.8)	22 (27.8)	
IVa	26 (8.1)	44 (7.7)	10 (7.7)	5 (6.3)	
b	0	3 (0.5)	0	0	
Presence of *SH*^3^ ova					
No	129 (26.8)	355 (40.7)	73 (46.5)	72 (58.1)	SCC < 0.0001
Yes	353 (73.2)	518 (59.3)	84 (53.5)	52 (41.9)	UC < 0.0001
Cystitis					
No	11 (6.7)	9 (2.8)	3 (4.7)	0	SCC 0.5
Yes	152 (93.3)	315 (97.2)	61 (95.3)	55 (100)	UC 0.2
Ulceration					
No	14 (11.2)	8 (3.3)	7 (15.6)	2 (2.7)	SCC 0.4
Yes	111 (88.8)	232 (96.7)	38 (84.4)	36 (97.3)	UC 0.8

^1^Comparison of *p* value between males and females was done using *χ*^2^; ^2^pelvic lymphadenectomy; ^3^*Schistosoma haematobium*.

**Table 3:** Unadjusted and adjusted (multivariable regression model) associations between male gender and different variables among squamous and urothelial cell carcinoma cases in Egypt.

Variable	Squamous cell carcinoma (SCC)		Urothelial cell carcinoma (UC)	
	OR (95% CI)^1^	AOR (95% CI)^2^	OR (95% CI)^1^	AOR (95% CI)^3^
Tumor grade				
1 + 2	Ref.	Ref.	Ref.	
3	1.64 (1.06–2.54)	0.82 (0.38–1.79)	0.99 (0.72–1.34)	
Presence of *SH*^4^ ova				
No	Ref.	Ref.	Ref.	Ref.
Yes	2.38 (1.64–3.45)	2.12 (1.15–3.89)	2.02 (1.38–2.96)	3.78 (1.89–7.55)
Age				
≤55 years	Ref.	Ref.	Ref.	Ref.
>55 years	2.25 (1.63–3.12)	2.40 (1.32–4.36)	1.31 (0.95–1.79)	3.44 (1.73–6.83)
Smoking				
Never	Ref.	Ref.	Ref.	Ref.
Ever	43.99 (16.97–114.05)	24.89 (8.58–72.21)	106.00 (30.28–371.10)	40.55 (9.13–180.12)
Residence				
North	Ref.	Ref.	Ref.	
South	2.20 (1.30–3.73)	3.31 (1.14–9.69)	0.94 (0.61–1.45)	

^1^Unadjusted odds ratio (95% confidence interval); ^2^adjusted odds ratios (95% confidence interval) in the multivariable model that included the variables listed in the left column in addition to education and secondhand smoke; ^3^adjusted odds ratios (95% confidence interval) in the multivariable model that included the variables in the left column (except residence and tumor grade), education, and secondhand smoke; ^4^*Schistosoma haematobium*; whenever smoking was assessed, pack-year for ever-smokers was included in the model.

## References

[1] Antoni S., Ferlay J., Soerjomataram I., Znaor A., Jemal A., Bray F. (2017). Bladder cancer incidence and mortality: a global overview and recent trends. *European Urology*.

[2] Fajkovic H., Halpern J. A., Cha E. K. (2011). Impact of gender on bladder cancer incidence, staging, and prognosis. *World Journal of Urology*.

[3] International Bladder Cancer Nomogram Consortium, Bochner B. H., Kattan M. W., Vora K. C. (2006). Postoperative nomogram predicting risk of recurrence after radical cystectomy for bladder cancer. *Journal of Clinical Oncology*.

[4] Welty C. J., Sanford T. H., Wright J. L. (2017). The Cancer of the Bladder Risk Assessment (COBRA) score: estimating mortality after radical cystectomy. *Cancer*.

[5] Shariat S. F., Favaretto R. L., Gupta A. (2011). Gender differences in radical nephroureterectomy for upper tract urothelial carcinoma. *World Journal of Urology*.

[6] IARC Working Group on the Evaluation of Carcinogenic Risks to Humans (2004). *Tobacco Smoke and Involuntary Smoking*.

[7] IARC Working Group on the Evaluation of Carcinogenic Risks to Humans (1994). *Schistosomes, Liver Flukes and Helicobacter pylori*.

[8] Silverman D. T., Devesa S. S., Moore L. E., Rothman N., Schottenfeld D., Fraumeni J. F. J. (2006). Bladder cancer. *Cancer Epidemiology and Prevention*.

[9] Al-Zalabani A. H., Stewart K. F., Wesselius A., Schols A. M., Zeegers M. P. (2016). Modifiable risk factors for the prevention of bladder cancer: a systematic review of meta-analyses. *European Journal of Epidemiology*.

[10] Burger M., Catto J. W., Dalbagni G. (2013). Epidemiology and risk factors of urothelial bladder cancer. *European Urology*.

[11] Hemelt M., Yamamoto H., Cheng K. K., Zeegers M. (2009). The effect of smoking on the male excess of bladder cancer: a meta-analysis and geographical analyses. *International Journal of Cancer*.

[12] Miyamoto H., Yang Z., Chen Y. T. (2007). Promotion of bladder cancer development and progression by androgen receptor signals. *Journal of the National Cancer Institute*.

[13] Hsu J. W., Hsu I., Xu D. (2013). Decreased tumorigenesis and mortality from bladder cancer in mice lacking urothelial androgen receptor. *The American Journal of Pathology*.

[14] Daugherty S. E., Lacey J. V., Pfeiffer R. M., Park Y., Hoover R. N., Silverman D. T. (2013). Reproductive factors and menopausal hormone therapy and bladder cancer risk in the NIH-AARP Diet and Health Study. *International Journal of Cancer*.

[15] McGrath M., Michaud D. S., De Vivo I. (2006). Hormonal and reproductive factors and the risk of bladder cancer in women. *American Journal of Epidemiology*.

[16] Cantwell M. M., Lacey J. V., Schairer C., Schatzkin A., Michaud D. S. (2006). Reproductive factors, exogenous hormone use and bladder cancer risk in a prospective study. *International Journal of Cancer*.

[17] Prizment A. E., Anderson K. E., Harlow B. L., Folsom A. R. (2007). Reproductive risk factors for incident bladder cancer: Iowa Women’s Health Study. *International Journal of Cancer*.

[18] Wolpert B. J., Amr S., Ezzat S. (2010). Estrogen exposure and bladder cancer risk in Egyptian women. *Maturitas*.

[19] Morales E. E., Grill S., Svateka R. S. (2016). Finasteride reduces risk of bladder cancer in a large prospective screening study. *European Urology*.

[20] Gupta P., Jain M., Kapoor R., Muruganandham K., Srivastava A., Mandhani A. (2009). Impact of age and gender on the clinicopathological characteristics of bladder cancer. *Indian Journal of Urology*.

[21] Horstmann M., Witthuhn R., Falk M., Stenzl A. (2008). Gender-specific differences in bladder cancer: a retrospective analysis. *Gender Medicine*.

[22] Scosyrev E., Noyes K., Feng C., Messing E. (2009). Sex and racial differences in bladder cancer presentation and mortality in the US. *Cancer*.

[23] Mallin K., David K. A., Carroll P. R., Milowsky M. I., Nanus D. M. (2011). Transitional cell carcinoma of the bladder: racial and gender disparities in survival (1993 to 2002), stage and grade (1993 to 2007). *The Journal of Urology*.

[24] Mungan N. A., Kiemeney L., van Dijck J., van der Poel H. J., Witjes J. A. (2000). Gender differences in stage distribution of bladder cancer. *Urology*.

[25] Otto W., May M., Fritsche H. M. (2012). Analysis of sex differences in cancer-specific survival and perioperative mortality following radical cystectomy: results of a large German multicenter study of nearly 2500 patients with urothelial carcinoma of the bladder. *Gender Medicine*.

[26] Zheng Y. L., Amr S., Saleh D. A. (2012). Urinary bladder cancer risk factors in Egypt: a multicenter case-control study. *Cancer Epidemiology Biomarkers & Prevention*.

[27] Amr S., Loffredo C. A., McClain K. M., Kallakury B., Zheng Y. L. (2015). Adenocarcinoma of the urinary bladder in Egypt: potential risk factors. *World Journal of Nephrology and Urology*.

[28] Amr S., Dawson R., Saleh D. (2014). Agricultural workers and urinary bladder cancer risk in Egypt. *Archives of Environmental & Occupational Health*.

[29] Kamat A. M., Bağcıoğlu M., Huri E. (2017). What is new in non-muscle-invasive bladder cancer in 2016?. *Turkish Journal of Urology*.

